# Validation of the Malay Version of the COVID-19 Anxiety Scale in Malaysia

**DOI:** 10.21315/mjms2022.29.3.12

**Published:** 2022-06-28

**Authors:** Chin Siang Ang, Kai Wei Lee, Meng Chuan Ho, Siok Ping Voon, Siew Mooi Ching, Chai Eng Tan, Fan Kee Hoo, Pei Boon Ooi

**Affiliations:** 1 School of Psychology, TMC Academy, Singapore; 2 Lee Kong Chian School of Medicine, Nanyang Technological University, Singapore; 3 Department of Pre-Clinical Sciences, Faculty of Medicine and Health Sciences, Universiti Tunku Abdul Rahman, Selangor, Malaysia; 4 Centre for Research on Communicable Diseases, Universiti Tunku Abdul Rahman, Selangor, Malaysia; 5 Department of General Studies, Faculty of Social Sciences and Liberal Arts, UCSI University, Kuala Lumpur, Malaysia; 6 Programme of Psychology, Faculty of Cognitive Sciences and Human Development, Universiti Malaysia Sarawak, Sarawak, Malaysia; 7 Department of Family Medicine, Faculty of Medicine and Health Sciences, Universiti Putra Malaysia, Selangor, Malaysia; 8 Malaysian Research Institute on Ageing, Universiti Putra Malaysia, Selangor, Malaysia; 9 Department of Medical Sciences, School of Medical and Life Sciences, Sunway University, Selangor, Malaysia; 10 Centre for Research, Bharath Institute of Higher Education and Research, Tamil Nadu, India; 11 Department of Family Medicine, Faculty of Medicine, Universiti Kebangsaan Malaysia, Kuala Lumpur, Malaysia; 12 Department of Medicine, Faculty of Medicine and Health Sciences, Universiti Putra Malaysia, Selangor, Malaysia

**Keywords:** COVID-19 Anxiety Scale, Malaysia, Malay, reliability, validation, psychometric properties

## Abstract

**Background:**

Malaysians are disillusioned with the increased number of COVID-19 infection cases and the prolonged lockdown period. As a result of COVID-19 mitigation measurements, Malaysians are experiencing emotional and psychological consequences such as anxiety. Thus, there is an urgent need to have an instrument that could serve as a tool to assess the psychological impact of COVID-19 among Malaysians rapidly.

**Methods:**

This study aimed to adapt and validate the Malay version of the COVID-19 Anxiety Scale (M-CAS) among Malaysian adults. The back-to-back translation was done to produce a M-CAS. Following face validation, M-CAS was self-administered to 225 participants from Malaysia via an online survey. The participants also completed the Generalised Anxiety Disorder 7-item Scale (GAD-7), World Health Organization Quality of Life Scale, Abbreviated Version (WHOQOL-BREF) and the Fear of COVID-19 Scale (FCV-19S). Data analysis was conducted using Statistical Package for the Social Sciences and Analysis of a Moment Structures. The psychometric properties of the M-CAS were examined via Cronbach alpha and confirmatory factor analysis. M-CAS scores were compared with the other tools to provide external validity.

**Results:**

The statistical analysis revealed that the M-CAS demonstrated adequate internal consistency (Cronbach’s alpha = 0.890) and presented with a unidimensional factor structure. M-CAS scores were strongly correlated with GAD-7 (*r* = 0.511, *P* < 0.001) and FCV-19S (*r* = 0.652, *P* < 0.001). Lack of correlation between M-CAS and WHOQOL-BREF showed that M-CAS scores did not reflect perceived quality of life.

**Conclusion:**

The M-CAS is a valid and reliable tool in the Malay language that can be self-administered among Malaysians to assess anxiety levels induced by COVID-19. The M-CAS has only 7 items and utilised little time in real-life clinical practice.

## Introduction

The year 2020 was an incredibly challenging year when the World Health Organization (WHO) declared novel coronavirus (COVID-19) a pandemic. The pandemic was like a wildfire expanding its negative impact on the world (1–2) and continues to damage global development, leaving many countries suffering socially, economically and psychologically (3).

Malaysia has been significantly affected by the pandemic. Since March 2020, the Malaysia government had taken a radical proactive approach to prevent the spreading of the virus by implementing the government-imposed social restrictions or lockdown; known as the Movement Control Order (MCO), by prohibiting mass movements and gatherings at all places nationwide to control the spread of the virus. However, the MCOs have brought an undeniable psychological impact on society worldwide in various aspects. The disruption and decisive nature of prolonged lockdown not only impacted the economy (4), but the action that required most companies to practice a ‘work from home’ policy also generated a sense of employment uncertainty, as well as feelings of isolation and loneliness among most people (5–6). Heavy dependency on social media for work, communication and information about COVID-19 also created the feeling of being overwhelmed or stressed about much of the COVID-19 related news, leading to anxiety and emotional fluctuations in daily life (7–8).

The Malaysian people are at a point of disillusionment. There is widespread fear, apprehension and worry among the general population. As of 28 July 2021, 1,061,476 COVID-19 cases and 8,551 deaths had been recorded (9). In recent weeks, the situation in Malaysia has worsened even after another nationwide lockdown imposed on 1 July 2021 (10). The news spreading around Malaysia about COVID-19 is of concern to many Malaysians more than usual. Hundreds of contract doctors in Malaysia’s state hospitals recently went on strike due to a lack of job security (11). The ongoing COVID-19 epidemic will have a long-term impact on mental health, as the uncertainty of the outbreak and the economic downturn will cause stress levels to increase. In the absence of clear expectations about when things will improve, exposure to these stressors and the implications of the epidemic may lead to anxiety.

Research has been conducted on the consequences of the COVID-19 pandemic (12). However, few instruments have been developed or adapted to evaluate the psychological impacts of COVID-19 (13–15). Specifically, no single approach has been validated for the Malaysian context suitable for measuring COVID-19 anxiety. Hence, this study aimed to adapt and validate the Malay version of the COVID-19 Anxiety Scale (M-CAS) for the Malaysian people.

## Methods

Study participants were recruited through convenience sampling to complete an anonymous online survey. Eligible participants were individuals aged 18 years old or older, able to read and understand the Malay language. Participants who could not understand the Malay language or provide consent or with any psychological or neurological diagnosis were excluded. A detailed description of the study’s goals and objectives was posted on social media platforms and personal networks and sent through e-mail. A Google Forms link and directions to complete the survey were also included in the recruitment notices. A snowballing method to increase the number of participants was used, whereby participants were encouraged to forward the survey to their professional and personal networks. A total of 225 Malaysians completed the online versions of the M-CAS with other validated Malay language scales as described below. Participant sociodemographic data were also collected. Institutional ethical approvals were obtained and all participants provided their informed consent via the online survey.

### Measures

The study tool to be validated in this study was the COVID-19 Anxiety Scale (CAS).

### Sociodemographic Variables

Several characteristics, including age, ethnicity and education level, were examined.

### COVID-19 Anxiety Scale

The CAS was developed by Silva, de Sampaio Brito and Pereira (16) to measure anxiety levels due to COVID-19. The scale includes seven items with four levels of agreement: i) not applicable to me (level 0); ii) rarely applicable to me (level 1); iii) sometimes applicable to me (level 2) and iv) very applicable to me (level 3). The higher the CAS score, the greater the level of anxiety about COVID-19. CAS reported good internal consistency (α = 0.890, ω = 0.890) (17).

### Criterion for Validity

The criterion for the validity of the CAS was evaluated using the following tools.

### Generalised Anxiety Disorder Scale

Using the Generalised Anxiety Disorder 7-item Scale (GAD-7) (18), validated for use in Malaysian populations by Mohd Sidik, Aroll and Goodyear-Smith (19), to measure their anxiety level. The original study reported Cronbach’s alpha of 0.740. The Malay version of the GAD-7 consists of seven items, each scored on a 4-point scale ranging from 0 (not at all) to 3 (nearly every day). Higher scores indicated higher levels of generalised anxiety disorder. In this study, the Cronbach’s alpha coefficient of the GAD-7 was 0.911. The GAD-7 was used in this study to assess the convergent validity of COVID-19 related anxiety. The M-CAS was anticipated to exhibit a moderate-to-strong positive correlation with the GAD-7.

### World Health Organization Quality of Life Scale, Abbreviated Version

The World Health Organization Quality of Life Scale, Abbreviated Version (WHOQOL-BREF) (WHOQOL Group) (20) was validated in the Malaysian populations by Hasanah, Naing and Rahman (21) and measures individuals’ level of satisfaction with their quality of life in four aspects:

- *Physical health*, related to self-rated health and physical status, is often understood as the absence of disease
- *Psychological health*, which measures the individual’s appraisal of their own life and the quality of their positive and negative emotions
- *Social relationships*, which evaluates the individual’s social relationships with others
- *Environment*, which assesses the individual’s participation in their current environment (21)

There are 26 items in the WHOQOL-BREF Malay version, rated on a 5-point scale ranging from 1 (very poor/never) to 5 (very good/always). Higher scores indicate a better quality of life. The Malay WHOQOL-BREF reported good internal consistency with Cronbach alpha ranging from 0.64 to 0.80 for the subscales (21). In the present study, Cronbach’s alpha coefficient was 0.646 for physical health, 0.666 for psychological health, 0.721 for social relationships and 0.869 for the environment. The WHOQOL-BREF was used to assess the discriminant validity of the M-CAS. This was based on the conceptual differences between anxiety and quality of life. The M-CAS was anticipated to exhibit a negative correlation or no correlation with the four subscales of the WHOQOL-BREF.

### Fear of COVID-19 Scale

Fear of COVID-19 Scale (FCV-19S) (13) was validated for use in Malaysian populations by Pang et al. (22) to measure the fear COVID-19. The Malay version of the FCV-19S consists of 7 items, each scored on a 5-point scale ranging from 1 (strongly disagree) to 5 (strongly agree). Higher scores indicate higher levels of fear toward COVID-19. In the previous study, FCV-19S reported good internal consistency and reliability with Cronbach alpha of 0.893 (22); the Cronbach alpha value was reported at α = 0.911 for this study. The FCV-19S was used to test the concurrent validity of the M-CAS. The M-CAS was anticipated to exhibit a moderate-to-strong positive correlation with the FCV-19S.

### Procedure

This study had three phases: i) translating the CAS into the Malay language; ii) piloting the pre-final version of the translated questionnaires and iii) conducting a validation study. Data were collected between 11 June 2021 and 10 July 2021.

#### Phase 1: Translation of the scales

The original author granted permission to translate and adapt the scale. The CAS underwent forward and backward translation (23). To maintain the conceptual and semantic equivalence of the items, the forward translation was performed by two independent bilingual professional certified translators. The Malay translations were reviewed by the research team and reconciled into a single Malay version. Minor changes were made in the wording of some items (e.g. the use of the word *bersantai* which carried the semantic of ‘relax’ instead of *berehat*, was used) to ensure that the translated version maintained the meaning of the original questionnaire and suited the local language. The reconciled Malay version was then back-translated into English by two more bilingual independent professional certified translators who did not have prior knowledge of the original scale to avoid biases. The back-translated versions were reviewed against the original English version to ensure that contextual meaning was preserved. Following this, content experts reviewed the translated CAS to determine the scale’s content validity. Content validity refers to the degree to which the instrument’s content adequately reflects the construct being measured (24). Domain experts were selected based on expertise, specialised training or work experience in the relevant fields.

We recommended that at least three experts be involved in the content validation process (24). The experts were asked to rate the relevance of each item to the measured domain on the recommended 4-point scale: 1 = not relevant, 2 = somewhat relevant, 3 = quite relevant and 4 = very relevant. The content validity index (CVI) was calculated to quantify the experts’ views. In this study, the CVI was divided into an item-level content validity index (I-CVI) and scale-level content validity index (S-CVI) (25). The I-CVI was calculated as the number of content experts who rated relevance as 3 or 4 divided by the total number of expert judgements. The S-CVI was the average of all I-CVIs divided by the number of items. In addition, experts were asked to provide their subjective assessments of the items. A minimum score of 0.78 for I-CVI and 0.80 for S-CVI are recommended for reproducing content validity (24).

The scores for content validity were assessed by six experts: i) a psychologist; ii) a medical science researcher; iii) a counsellor and iv) three medical specialists. Of the six experts, four were women and two were men; all had extensive professional experience and were knowledgeable in their respective fields appropriate for this study. The I-CVI was 1.00 for all items except item 1 (‘I feel bad when thinking about COVID-19’). However, the score still met the criterion for content validity (0.83). The S-CVI was 0.98; therefore, all seven items were relevant to the respective construct.

However, based on the experts’ comments and suggestions, some improvements were made to the items of the instruments. The feedback from the experts included words that cannot be translated into Malay. Examples include:

- Update myself about COVID-19: the translators suggested *mengemaskini diri dengan COVID-19* but the experts suggested *mengikuti perkembangan terbaru COVID-19*
- Trouble relaxing: the translators suggested *menghadapi masalah untuk bersantai* but the experts suggested *sukar bersantai*
- Choose a better word (i.e. feel bad): the translators suggested *berasa tidak enak* or *berfikiran buruk* but the experts suggested *berasa tidak sedap hati*
- Revise the instruction for clarity (i.e. in the last few days): the translator suggested *dalam beberapa hari yang lepas* but the experts suggested *dalam beberapa hari kebelakangan ini*

#### Phase 2: Pre-testing

Face validation was conducted after content validation to check for clarity of instructions and language, whether ambiguous or unclear. Face validity refers to how well a test appears to measure what it is supposed to measure (25). The translated scale was distributed to a group of university students to check the clarity of the items. A minimum of 10 participants is recommended for face validation (25). Participants were asked to rate the clarity of each item on the recommended 4-point scale: 1 = not clear, 2 = somewhat clear, 3 = fairly clear and 4 = very clear. The face validity index (FVI) was calculated to quantify the views of the student participants. This study divided FVI into an item-level face validity index (I-FVI) and a scale-level face validity index (S-FVI). The I-FVI was the number of content experts who rated clarity as 3 or 4 divided by the total number of student participants. The S-FVI was the average of all I-FVIs divided by the number of items. Because there was no consensus on FVI cutoffs, we used the criterion for content validity (25). An I-FVI of 0.78 or higher and an S-FVI of 0.8 or higher were considered good face validity. Additionally, participants were asked to provide written comments on any items that seemed unclear to them.

The results for face validity were assessed by 15 university students. Of these, six were women and nine were men; all were between the ages of 18 years old and 24 years old. Ten of them were diploma students and five were undergraduates. The I-FVI was 1.00, so the S-FVI was also 1.00. The statistics show that all seven items were measured as intended. Overall, the participants described the scale as understandable and straightforward, with a couple of exceptions. After receiving their feedback, additional wording changes were made, for example, *Saya berasa tidak senang hati ketika memikirkan COVID-19* to *Saya berasa tidak senang hati apabila memikirkan COVID-19* as the word *apabila* reflected the meaning of ‘about’ instead of *ketika* which reflected ‘while’ (Table 1 for each item in Malay and the item in English from which it was translated). The final version of the M-CAS was easy to read and understand during pre-testing and was ready for the construct validation process.

#### Phase 3: Validation of the Malay scale

The newly translated scale was subsequently assessed for its psychometric properties, including its validity and reliability, in a large-scale validation study. The required sample size for this phase was 170 participants, based on the recommendation of 1:10 ratios of items to participants (26).

## Data Analysis

Data were analysed using the IBM SPSS version 26.0 and IBM AMOS 20.0 statistical analysis systems. Before conducting the CAS, the seven CAS items were checked for missing values and normality. No values were missing. Univariate and multivariate normality was assessed. Based on the unidimensional factor structure of the original CAS, confirmatory factor analysis (CFA) was used to evaluate its structural validity, with bootstrap maximum likelihood estimation of 2,000 samples. Because the chi-squared statistic is highly sensitive to sample size, several goodness-of-fit indices were considered: the comparative fit index (CFI) (27), the Tucker-Lewis index (TLI) (28), bootstrap maximum likelihood estimation with 2,000 samples and the Root Mean Square Error of Approximation (RMSEA) (29). For CFI and TLI, values ≥ 0.90 are acceptable (29) whereas, Standardised Root Mean Square Residual (SRMR) ≤ 0.08 (29) and RMSEA ≤ 0.1 (30) are acceptable. Pearson’s correlation coefficient was used to test convergent validity, discriminant validity and concurrent validity with a series of previously validated scales. For internal consistency, we used corrected item-total correlations within the range of 0.30 to 0.80 and a reliability coefficient of 0.70 and above (31).

## Results

### Demographic Profile

A total of 225 Malaysians participated, with more women (*n* = 154, 68.4%) participating than men. Participants were aged 18 years old–70 years old, with a mean age of 26.18 (SD 9.46); three participants preferred not to reveal their ages. There were more participants of Chinese ethnicity (*n* = 102, 45.3%), followed by Malay (*n* = 95, 42.2%), Indian (*n* = 18, 8.0%) and others (*n* = 10, 4.5%). Most of the participants were highly educated, with about 89.6% of participants possessing post-secondary education (diploma, bachelor’s degree or postgraduate degrees), whereas 10.4% completed only primary or secondary school.

### Item Properties and Inter-Item Correlations

The skewness and kurtosis measures were used to examine univariate normality. Some items were found to be outside the normal range. The distribution of M-CAS items showed a slight negative skewness. To be considered acceptable, multivariate normality must be less than 5 (32). If the multivariate kurtosis values of M-CAS exceeded the value, the multivariate normality assumption was not valid. Because normality is an essential assumption of structural modeling, we used bootstrapping for CFA (32). All results are presented in Table 2.

The M-CAS had inter-item correlation coefficients ranging from 0.254 to 0.707 at *P* < 0.001. A moderate-to-high correlation among scale items could be interpreted as evidence of unidimensionality, indicating that the test measures only one trait. In making inferences, it is, therefore, necessary to inspect factorial construct validity.

### Factorial Construct Validity

**Figure 1:**
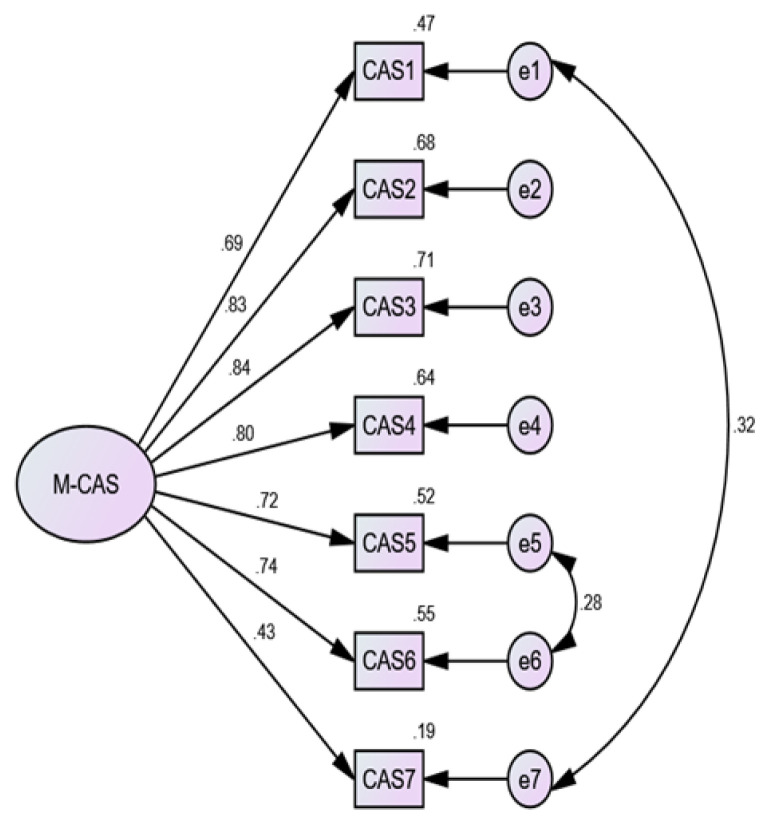
Standardised factor loading for the M-CAS

Regarding the factor structure of the M-CAS, a one-factor model was evaluated in which all items were loaded on one factor. All fit indexes met their respective criteria except RMSEA (Table 3). The model was significantly improved in its fit by adjusting the error terms of items 1 and 7, and items 5 and 6 (to produce M1a). There was a statistical significance for all items with a critical ratio > 1.96 and above the 0.40 cutoff (32). The magnitude ranged from 0.430 to 0.840 (Figure 1). The findings suggested that the M-CAS had a one-dimensional factor structure similar to the original English version.

### Convergent, Discriminant and Concurrent Validity

The correlations between these scales follow the expected pattern (Table 4). There were significant positive correlations between the M-CAS with the GAD-7, which provided evidence of convergent validity. There were no significant correlations between the M-CAS with the subscales of the WHOQOL-BREF, indicating they had discriminant validity. In addition, the M-CAS exhibited moderate-to-strong positive correlations with the FCV-19S; concurrent validity was thus supported.

### Internal Consistency

As shown in Table 5, the M-CAS showed significant homogeneity. The corrected item-total correlation coefficients for the M-CAS ranged between 0.445 and 0.772. The M-CAS had an alpha coefficient of 0.890, which demonstrated good internal consistency.

## Discussion

This study examined the psychometric properties and validity of M-CAS. Overall, the anxiety level reported by the participants was considered low, with 5 of the 7 items having mean scores of less than 2.0. This could be because, at the point of data collection (June 2021–July 2021), Malaysia was in the midst of the national immunisation programmes (NIP) where Malaysians would be getting their COVID-19 vaccination. Despite a slow rollout rate in February 2021, Malaysia’s vaccination rate increased steadily in June, with private hospitals and general practitioner clinics’ involvement, resulting in a higher distribution and availability of the vaccine (33). This increase may have lessened anxiety levels among Malaysians.

The translated measure (M-CAS) was found to have conceptual and semantic equivalence to the original English measures, providing evidence of linguistic validity. Pre-testing of the M-CAS provided face validity for the tool because it was easy to understand. The scale had good internal consistency reliability. CFA supported the unifactorial structure for the scale. The scale was also convergent, discriminant, and concurrently valid with various established psychological measures for anxiety and quality of life.

In this study, the M-CAS exhibited excellent internal consistency in which the Cronbach’s alpha coefficient was 0.890, similar to the original English version (16). The decision to translate, adapt and validate the CAS by Silva et al. (17) was based on the tool’s ability to evaluate anxiety specific to the context of COVID-19. This study adopted the CAS instead of a similar tool, the Coronavirus Anxiety Scale by Lee (34). Although Lee’s Coronavirus Anxiety Scale was found to have good internal consistency reliability for evaluating dysfunctional anxiety related to the coronavirus pandemic, the CAS by Silva et al. (17) better reflected the symptoms of anxiety following the classification outlined in the Diagnostic and Statistical Manual of Mental Disorders (DSM-5) (35), which encapsulated the emotion of fear and feeling of uneasiness.

Additionally, the tool was also sensitive to gauge fluctuations in the level of COVID-19 anxiety with a 4-point rating scale ranging from 0 (not applicable to me) to 3 (very applicable to me) (16). The CAS was developed in Brazil, which also reported an increase in COVID-19 infection and death rate, the same situation faced in Malaysia. The similarity of the context gives it additional value for adaptation in our local setting with the wording of the items applying to the local population. Furthermore, the CAS has yet to be validated in any foreign language; this study will be among the first.

More importantly, the results of the CFA yielded statistically significant and relatively high factor loadings, further indicating that the translated items were adequate. A similar finding was also seen in the English version of CAS (16), in which the factor loading for most of the items adequately exceeded the threshold; the exception was item 7, which was 0.40 in the CFA and 0.53 in the exploratory factor analysis *(*EFA). Overall, based on the result of several goodness fit indices in CFA, M-CAS was shown to be a unidimensional factor structure (*χ*^2^/df ≤ 5, TLI and CFI > 0.9 and RMSEA < 0.1) (36). The unidimensionality of the M-CAS is consistent with the Silva, de Sampaio Brito and Pereira’s (16) CAS English version.

Convergent validity shows that items in the scale that should measure the same construct do indeed measure the same construct. Therefore, M-CAS, which aims to measure anxiety related to COVID-19, should demonstrate a positive correlation with another scale that has been validated to measure anxiety, such as the GAD-7 (36). It should also show a positive correlation with FCV-19S, which measures fear. Anxiety and fear are closely related psychological concepts; thus, the M-CAS strongly correlated with the FCV-19 scores.

In the current context, M-CAS possessed a moderate-to-strong correlation (*r* = 0.511, *P* < 0.001) with GAD-7 and with FCV-19S (*r* = 0.652, *P* < 0.001). This proves that M-CAS does indeed measure anxiety. Concurrent validity was demonstrated by M-CAS scores positively correlated significantly with FCV-19S (*r* = 0.652, *P* < 0.001). This indicated that M-CAS is capable of predicting the fear of COVID-19.

Discriminant validity is demonstrated when scales that do not measure the same construct are not correlated with one another. In this study, WHOQOL-BREF measured perceived quality of life, which is conceptually different from anxiety. Therefore, the M-CAS scores should not correlate with the WHOQOL-BREF scores. The lack of a significant correlation between the M-CAS and the WHOQOL-BREF demonstrates discriminant validity for the M-CAS.

## Strengths and Limitations

This study adopted a rigorous method in the translation and adaptation of the tool for the Malaysian population. The M-CAS could be a tool for further research. It could also be made accessible to healthcare providers to assess and identify people at risk for anxiety concerning COVID-19, thus allowing for early and on-time intervention.

However, the M-CAS was assessed among participants with post-secondary education. The suitability of using this tool among those with lower educational status needs to be re-evaluated. Additionally, the demographic characteristics, particularly on ethnicity distribution, did not reflect the actual demographics of Malaysia. These were limitations due to the convenience sampling method used for this study because of pandemic-related social restrictions. Therefore, the findings of this study should be interpreted with caution in terms of generalisability because of the sociodemographic profile of the study respondents. However, statistical methods were used to prove the validity of M-CAS, which was established to have sound and valid psychometric properties for adoption.

Future studies should assess the validity of the M-CAS among a general adult Malaysian population with respondents from different socioeconomic statuses. The M-CAS can be used to evaluate COVID-19 related anxiety among the general population, particularly among those with at least secondary to post-secondary educations.

## Conclusion

This study contributes a Malay version of the CAS to the literature and demonstrates that it meets the psychometric criteria for its use in Malay-speaking populations. Recent research suggests that Malaysians’ mental health has been affected by COVID-19 due to their health and financial status concerns. Therefore, it is hoped that the availability of this scale will promote psychological research related to COVID-19 anxiety in Malaysia. Furthermore, the M-CAS is quite easy to use and requires little time in clinical practice.

## Figures and Tables

**Table 1:** Comparison of the original English CAS and Malay M-CAS items

Item	Original English version	Malay version
1	I feel bad when thinking about COVID-19	*Saya berasa tidak senang hati apabila memikirkan COVID-19*
2	I feel heart racing when I read about COVID-19	*Saya berasa jantung saya berdebar apabila saya membaca tentang COVID-19*
3	I feel anxious about COVID-19	*Saya berasa resah dengan COVID-19*
4	I feel uneasy when reading news about COVID-19	*Saya berasa tidak selesa apabila membaca berita tentang COVID-19*
5	I have trouble relaxing when I think about COVID-19	*Saya sukar bersantai apabila memikirkan COVID-19*
6	I feel like I may panic when I update myself about COVID-19	*Saya mungkin berasa panik apabila saya mengikuti perkembangan terbaru Covid-19*.
7	I am afraid of being infected with COVID-19	*Saya takut dijangkiti COVID-19*

**Table 2:** Mean scores for the M-CAS items and their distribution parameters

Scale	Mean	SD	Skewness	Skewness ratio	Kurtosis	Kurtosis ratio
M-CAS						
Item 1	2.05	0.830	−0.517	−3.191	−0.402	−1.245
Item 2	1.37	0.913	0.043	0.265	−0.830	−2.570
Item 3	1.76	0.932	−0.315	−1.944	−0.753	−2.331
Item 4	1.66	0.917	−0.085	−0.525	−0.844	−2.613
Item 5	1.45	0.986	0.004	0.025	−1.020	−3.158
Item 6	1.38	0.984	0.204	1.259	−0.959	−2.970
Item 7	2.31	0.850	−0.942	−5.815	−0.131	−0.406
Multivariate					12.671	8.466

Notes: SD = standard deviation; Skewness ratio = skewness/standard error of skewness for each item; Kurtosis ratio = kurtosis/ standardised error of kurtosis for each item. A skewness ratio and kurtosis ratio larger than 1.96 can be regarded as signs of non-normality

**Table 3:** Summary of model fit indices for the M-CAS

Model	*χ* ^2^	df	CFI	TLI	SRMR	RMSEA (90% CI)
M-CAS						
M1: one factor	64.539	14	0.939	0.908	0.053	0.127 (0.097–0.159)
M1a: modified, one factor	27.778	12	0.981	0.967	0.031	0.077 (0.039–0.114)

Notes: χ^2^ = Chi-squared; df = degree of freedom; CFI = comparative fit index; TLI = Tucker-Lewis index; SRMR = Standardised Root Mean Square Residual; RMSEA = Root Mean Square Error of Approximation

**Table 4:** Criterion validity of M-CAS

Scale	Convergent validity	Discriminant validity				Concurrent validity
		
	GAD-7	WHOQOL-BREF				FCV-19S
	
		PhH	PsH	SR	Env	
M-CAS	0.511***	0.002	−0.069	0.014	−0.122	0.652***

Notes: GAD-7 = Generalised Anxiety Disorder 7-item Scale; WHOQOL-BREF = World Health Organization Quality of Life Scale; PhH = physical health; PsH = psychological health; SR = social relationship; Env = environment; FCV-19S = Fear of COVID-19 Scale;

*** *P* < 0.001

**Table 5:** Internal consistency of the M-CAS

Item	M-CAS	

	Corrected item-total correlation	Squared multiple correlation
Item 1	0.671	0.511
Item 2	0.757	0.608
Item 3	0.772	0.629
Item 4	0.734	0.584
Item 5	0.684	0.555
Item 6	0.732	0.567
